# Impact of the disruption in supply of varenicline since 2021 on smoking cessation in England: A population study

**DOI:** 10.1111/add.16485

**Published:** 2024-04-30

**Authors:** Sarah E. Jackson, Jamie Brown, Harry Tattan‐Birch, Lion Shahab

**Affiliations:** ^1^ Department of Behavioural Science and Health University College London London UK; ^2^ SPECTRUM Consortium Edinburgh UK

**Keywords:** Champix, Chantix, population study, quit attempts, smoking cessation, varenicline

## Abstract

**Background and aims:**

Varenicline is one of the most effective smoking cessation treatments. Its supply in England was disrupted in July 2021 due to nitrosamine impurities found by its supplier, Pfizer. This study measured the impact of this disruption on smoking cessation in England.

**Design, setting and participants:**

The study used repeated cross‐sectional surveys conducted monthly, from June 2018 to December 2022. Set in England, it comprised a total of 3024 adults who reported smoking during the past year and had made at least one serious attempt to quit in the past 6 months.

**Measurements:**

Generalized additive models analyzed the association of the varenicline supply disruption with the trend in self‐reported varenicline use in the most recent quit attempt. We used these results to estimate the population‐level impact of the disruption on smoking cessation.

**Findings:**

Before July 2021, the proportion of past 6‐month quit attempts using varenicline was stable at approximately 3.9% [risk ratio (RR)_trend_ = 1.034, 95% confidence interval (CI) = 0.823–1.298]. The trend in varenicline use has changed sharply since the supply disruption (RR_Δtrend_ = 0.297, 95% CI = 0.120–0.738), with prevalence falling by 69.3% per year since; from 4.1% in June 2021 to 0.8% in December 2022. Convergently, National Health Service general practitioner prescribing data reported that just 0.1% of prescriptions for smoking cessation treatments in December 2022 were for varenicline. Assuming that varenicline does not return to the market, we estimate that this could result in ~8400 fewer people stopping smoking for at least 6 months, ~4200 fewer long‐term ex‐smokers and ~1890 more avoidable deaths each year.

**Conclusions:**

In England, the disruption in supply of varenicline since 2021 has coincided with a substantial fall in the use of varenicline in attempts to quit smoking.

## INTRODUCTION

Tobacco smoking remains a leading cause of morbidity and mortality in England [1]. More than a third of people in England who smoke make a serious attempt to stop smoking each year [2], but most quit attempts do not result in long‐term abstinence [2]. Varenicline—a partial nicotinic acetylcholine receptor agonist—is one of the most effective smoking cessation treatments, especially when paired with behavioural support [3, 4, 5]. However, the supply of varenicline was disrupted in 2021, substantially reducing its availability for use by people in quit attempts. Understanding the impact of this disruption in supply upon the use of varenicline in quit attempts and implications for population‐level cessation is important for informing policy decisions around alternative treatments. For example, if the unavailability of varenicline reduced the number of people quitting each year, causing avoidable deaths, then policymakers may need to invest in and promote suitable alternatives (e.g. cytisine).

Pfizer, the sole supplier of varenicline in England to February 2023, paused the distribution of all varenicline products (0.5‐ and 1‐mg tablets, under the trade names Champix and Chantix) in June 2021 as a precaution after detecting the presence of nitrosamine impurities (N‐nitroso‐varenicline) above Pfizer's acceptable level of daily intake in several batches. Some nitrosamines may increase the risk of cancer if people are exposed to them above acceptable levels and over long periods of time [6, 7]. In their statement, Pfizer advised: ‘For those patients currently prescribed Champix we believe the benefits outweigh the very low potential risks, if any, posed by nitrosamine exposure from varenicline on top of other common sources over a lifetime’ [8]. At this stage, the Department of Health and Social Care issued a supply disruption alert (SDA/2021/006), but did not discourage the use of varenicline. Health‐care professionals and trained stop smoking advisers involved in delivering smoking cessation services were advised to review all patients currently prescribed varenicline, determine the availability of varenicline tablets and prescribe nicotine replacement therapy (NRT) as an alternative where supplies of varenicline were unavailable. For new patients, all providers of smoking cessation services were advised to avoid initiating varenicline treatment and to advise on alternative pharmacotherapy options (i.e. NRT and bupropion).

After the original supply disruption alert was issued on 24 June 2021, the European Medicines Agency Committee for Medicinal Products for Human Use (EMA CHMP) issued their decision on the acceptable limit of impurities to be present in varenicline products in the European Union, which required Pfizer to vary its licence to comply with the set requirements [9]. The UK Medicines and Healthcare products Regulatory Agency (MHRA) followed the CHMP's approach and initiated a class 2 medicines recall (EL 21 A/25) of all in‐date batches of varenicline at pharmacy and wholesaler level on 14 October 2021. An updated supply disruption alert (SDA/2021/006[U]) was issued on 28 October 2021, confirming that there was no re‐supply date of varenicline from Pfizer and no alternative supplier. Health‐care professionals were advised to return all remaining stock of the specified batches of varenicline to their supplier, to review patients currently prescribed varenicline and switch them to NRT unless contraindicated and not to initiate varenicline products for any new patients.

As of February 2024, there has been no news regarding when supplies might recommence in England, but it is unlikely to be in the short term. Generic varenicline is available in the United States, Canada and Australia, but a generic was not licensed and supplied in England up to February 2024. As varenicline was previously one of the most effective treatments available to people who smoke [3, 4, 5], it is plausible that this shortage has suppressed the success rate of quit attempts by compelling people to use less effective forms of support, and will continue to do so while the drug remains unavailable. In this study, we aimed to estimate the population impact of the disruption in supply of varenicline since 2021 on smoking cessation in England. Specifically, we addressed the following research questions:

1. What has been the impact of the varenicline supply disruption on monthly trends in varenicline use in people's attempts to quit smoking?
2. What is the probable population‐level impact of the varenicline supply disruption on cessation?

## METHOD

### Design

To estimate the association of the varenicline supply disruption with varenicline use we used data from the Smoking Toolkit Study (STS): a monthly representative household survey. The STS uses a hybrid of random probability and simple quota sampling to select a new sample of 1700 adults in England each month [10]. Comparisons with sales data and other national surveys indicate that key variables, including socio‐demographics, smoking prevalence and cigarette consumption, are nationally representative [10, 11]. We used data collected from June 2018 (3 years before Pfizer stopped the distribution of varenicline) to December 2022 (the most recent data available at the time of analysis). Data were initially collected through face‐to‐face computer‐assisted interviews. However, social distancing restrictions under the COVID‐19 pandemic meant that no data were collected in March 2020, and data from April 2020 onwards were collected via telephone. The telephone‐based data collection used a similar combination of random location and quota sampling and weighting approach as the face‐to‐face interviews. After social distancing restrictions were lifted, we ran parallel surveys using the two data collection modalities in the same month to evaluate the impact of the change in methods on key outcomes; the data yielded similar estimates for key socio‐demographic and smoking measures [12]. We limited our sample to people who had smoked during the past year (i.e. those who reported current smoking or who had quit during the past year) aged ≥ 18 years.

We then used the findings of our STS analysis, in combination with data from other sources on population size [13], smoking prevalence [14], the effectiveness of varenicline and other forms of pharmacotherapy for smoking cessation [3] and prescription of smoking cessation treatments in National Health Service (NHS) general practices (GP) in England [15]), to estimate the impact of the disruption in supply of varenicline on smoking cessation at the population level.

### Measures

#### Sample selection

Quit attempts were assessed with the question: ‘How many serious attempts to stop smoking have you made in the last 12 months? By serious attempt I mean you decided that you would try to make sure you never smoked again. Please include any attempt that you are currently making and please include any successful attempt made within the last year’. A follow‐up question asked about time since the most recent quit attempt began: ‘How long ago did your most recent serious quit attempt start? By most recent, we mean the last time you tried to quit’. We analyzed data from those who reported having made at least one quit attempt during the last 6 months. We selected past 6‐month quit attempts over past‐year attempts to provide a more sensitive estimate of recent changes and to reduce the risk of recall bias.

#### Outcome variable

Among those who reported trying to quit, use of varenicline was assessed with the question: ‘Which, if any, of the following did you try to help you stop smoking during the most recent serious quit attempt?’. Participants were asked to indicate all that apply. Those who responded ‘Champix (varenicline)’ were coded 1, otherwise they were coded 0.

##### Trends, effects of disruption in supply of varenicline and seasonality

Time was measured in months throughout the study period (coded 1… *n*, where *n* was the total number of waves), which allowed us to account for underlying secular trends. An additional variable reflected the change in trend (slope) following the disruption in supply (coded 0 before and 1… *m* from July 2021 onwards, where *m* was the number of waves after the disruption). We treated the timing of the interruption as July 2021 rather than June 2021, because the supply disruption alert was issued on 24 June 2021, near the end of the month and after the STS data for June were collected.

To control for seasonality (month‐of‐year effects), the month within the year (calendar month) was coded from January = 1 to December = 12. This allowed us to account for any regular seasonal pattern in the use of varenicline (see Statistical analysis).

##### Covariates

Socio‐demographic covariates were age, gender, occupational social grade and level of cigarette addiction. Age was analyzed as a continuous variable. Gender was self‐identified as man, woman or ‘in another way’; we excluded those identifying in another way from analyses that adjusted for gender due to low numbers. Occupational social grade was categorized as ABC1, which includes managerial, professional and intermediate occupations, and C2DE, which includes small employers and own‐account workers, lower supervisory and technical occupations and semi‐routine and routine occupations, state pensioners, never worked and long‐term unemployed. Level of cigarette addiction was assessed by self‐reported ratings of the strength of urges to smoke during the last 24 hours [not at all (coded 0), slight (1), moderate (2), strong (3), very strong (4), extremely strong (5)]. This question was also coded ‘0’ for those who responded ‘not at all’ to the (separate) question ‘How much of the time have you spent with the urge to smoke?’.

### Statistical analysis

These analyses were not pre‐registered and should be considered exploratory. Data were analyzed using R version 4.2.2. The STS uses raking to create survey weights that match the sample to the population in England on the dimensions of age, social grade, region, housing tenure, ethnicity and working status within sex [10]. All the following analyses were performed on weighted data. Missing cases were excluded on a per‐analysis basis.

We used segmented regression to assess the effect of the varenicline supply disruption on the use of varenicline in the most recent past 6‐month quit attempt. We used log‐binomial generalized additive models (GAMs, using the mgcv package in R) to model trends before the disruption in supply (underlying secular trend) and the change in the trend (slope) after the disruption (see Measures for details on how these trend variables were coded). We ran two models: model 1 was adjusted for seasonality (modelled using a smoothing term with cyclic cubic splines specified) and model 2 was adjusted for seasonality and covariates. We assumed a linear trend before the disruption in supply, based on the relatively short length of the time‐series (meaning that we expected negligible differences between log‐linear and linear trends). We used predicted estimates from the adjusted model to plot time trends in the weighted prevalence of use of varenicline alongside unadjusted, weighted quarterly data points.

We then used the results of these analyses to estimate the impact of the disruption in supply of varenicline on smoking cessation at the population level, Cact of e‐cigarettes on cessation [16].

## RESULTS

A total of 15 424 people in England who had smoked during the past year were surveyed in the STS between June 2018 and December 2022. Of these, 3024 reported having made at least one serious quit attempt in the past 6 months and formed our analytical sample. Participants had a weighted mean age of 38.2 [standard deviation (SD) = 15.3] years, 45.8% were female and 58.8% were from social grades C2DE. The mean rating of strength of urges to smoke (on a scale from 0 to 5) was 1.7 (SD = 1.2).

**FIGURE 1:**
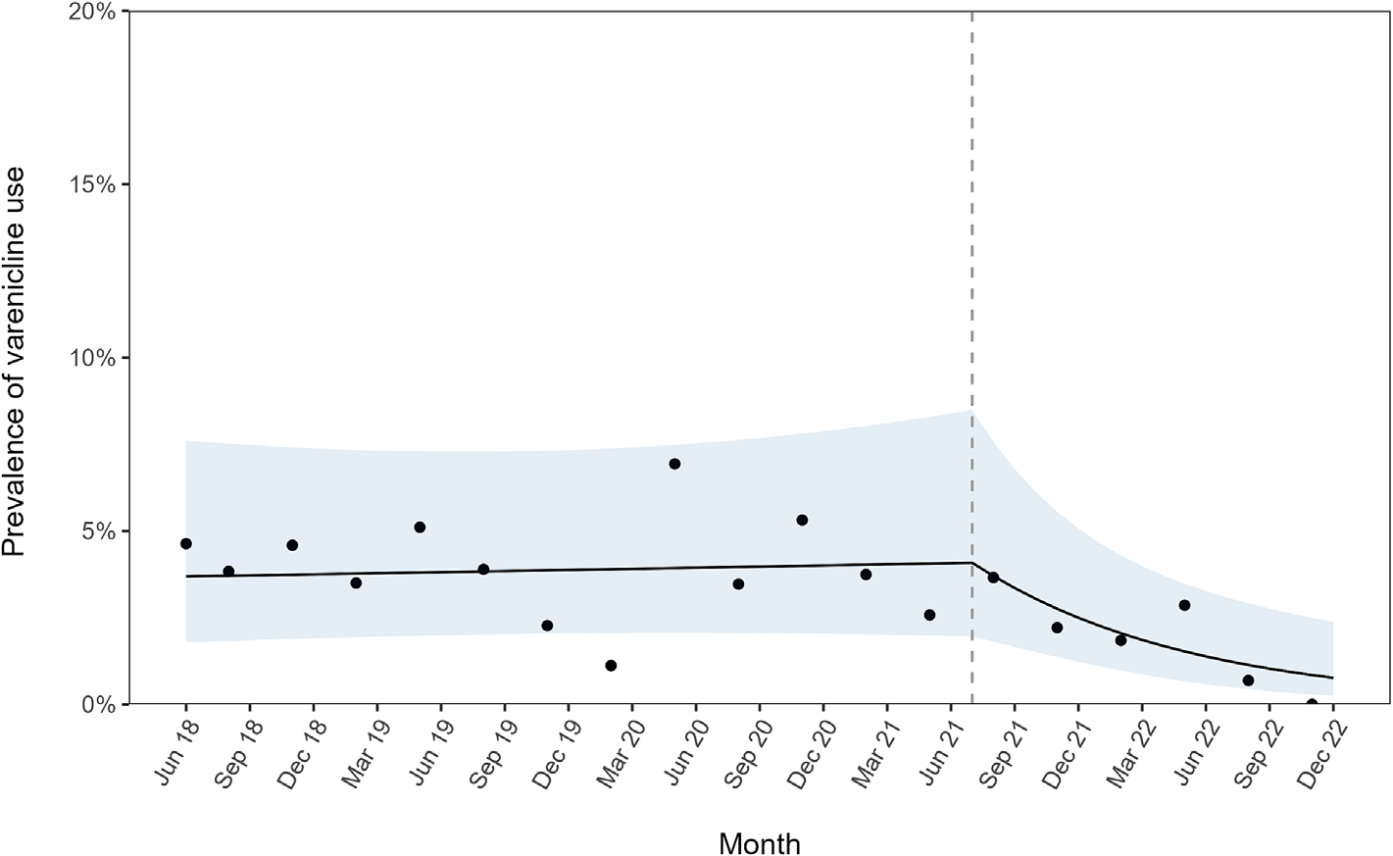
Trends in the prevalence of varenicline use, before and after the disruption in supply. The line represents modelled weighted prevalence of varenicline use in the most recent past 6‐month quit attempt over the study period (June 2018–December 2022), adjusted for seasonality, age, gender, social grade and strength of urges to smoke (coefficients shown in model 2, Table 1). Points represent unadjusted weighted prevalence by quarter. The vertical dashed line indicates the timing of the varenicline supply disruption in July 2021.

Table 1 summarizes the GAM results. There was a notable change in the trend in varenicline use by people who smoked during their most recent past 6‐month quit attempt following the disruption (Figure 1). Before July 2021, the proportion of quit attempts using varenicline was stable at approximately 3.9% [risk ratio (RR)_trend_ = 1.034, 95% confidence interval (CI) = 0.823–1.298]. The disruption in supply was associated with a substantial downward change in trend (RR_Δtrend_ = 0.297, 95% CI = 0.120–0.738), with prevalence falling by 69.3% per year since the disruption (RR_trend_ × RR_Δtrend_ = 1.034 × 0.297 = 0.307): from 4.1% in June 2021 to 0.8% in December 2022 (Figure 1). The unmodelled data showed that no participants surveyed in the last quarter of 2022 reported using varenicline in their most recent quit attempt (Figure 1).

**TABLE 1:** GAM results: associations of the varenicline supply disruption with use of varenicline in the most recent past 6‐month quit attempt.

	Model 1			Model 2		
	RR	95% CI	*P*	RR	95% CI	*P*
Used varenicline in most recent quit attempt^a^						
Trend (year)	0.985	0.790–1.230	0.897	1.034	0.823–1.298	0.776
ΔTrend (year)	0.324	0.134–0.779	0.012	0.297	0.120–0.738	0.009

*Note*: Model 1 was adjusted for seasonality. Model 2 was adjusted for seasonality, age, gender, social grade and strength of urges to smoke. Trend is the underlying secular trend. ΔTrend is the change in trend (slope) following the disruption in supply of varenicline.

Abbreviations: CI = confidence interval; RR = risk ratio; GAM = generalized additive models.

^a^ Among people who smoked in the past year and had tried to quit in the past 6 months (unweighted *n* = 3024).

### Estimating the population‐level impact on cessation

1. There are approximately 5.82 million adults who smoke in England (13.0% of 44.77 million people aged ≥ 18 years) [13, 14].
2. Of these, approximately 37% (~2.2 million people) attempt to quit each year [2].
3. Before July 2021, 3.9% of these were using varenicline to try to stop smoking each year [~85 800 people (STS analysis: mean modelled monthly prevalence, June 2018–June 2021)].
4. This has fallen to 2.1% since July 2021 (STS analysis: mean modelled monthly prevalence, July 2021–December 2022), and lower in more recent months [0.8% of past 6‐month quit attempts in December 2022 (STS analysis: modelled prevalence); 0% in Q4 2022 (STS analysis: unadjusted weighted prevalence)].
5. Prescription data from NHS GP practices in England corroborate this decline, showing that just 0.1% of prescriptions (35 of 29 112 items) for smoking cessation treatment in December 2022 were for varenicline [15]. Given that < 10% of people who try to stop smoking use prescription medication [17], the prescription data indicate that use of varenicline by those who tried to quit has fallen to virtually 0%.
6. Therefore, it is estimated that between 1.8% (3.9–2.1%; conservative estimate) and 3.9% (3.9–0%; upper limit) of people who have tried to stop smoking each year since July 2021 [between 39 600 (2 200 000 × 0.018) and 85 800 people (2 200 000 × 0.039)] did not use varenicline who otherwise would have if there were no supply disruption.
7. With varenicline not being on the market, if we assume that all these people moved over to other prescription medication (NRT or bupropion) this will have reduced the number of successful quit attempts, as varenicline is nearly 40% more effective than either NRT or bupropion [3]. The absolute difference in 6‐month abstinence rates for varenicline is ~6.9% higher than for NRT or bupropion [3]. This means that the disruption in supply of varenicline has resulted in between 2700 (39 600 × 0.069) and 5900 (85 800 × 0.069) fewer people stopping smoking for at least 6 months each year.
8. However, prescribing patterns in GP practices show that there was a 35% drop in the average number of monthly prescriptions for all smoking cessation medications, from 55 490 per month during the period from June 2018 to June 2021 to 35 899 during the period from July 2021 to December 2022 [15]. This suggests that while bupropion prescriptions have more than doubled (+140.9%, from an average of 1957, June 2018–June 2021 to 4715, July 2021–December 2022), this has not made up for the huge shortfall in varenicline prescriptions (NRT prescriptions have remained similar; +0.1%, from an average of 30 358 to 30 389, respectively) [15]. Previous analyses have shown that the increase in prevalence of the use of e‐cigarettes in quit attempts appeared unrelated to the prevalence of the use of prescription treatments [18, 19], so if we assume that 35% of those who would have used varenicline did not use another medication or an e‐cigarette, this would mean the reduction in 6‐month quits presented in step 7 of this calculation is an underestimate.
9. The absolute difference in 6‐month abstinence rates for varenicline is ~15% higher compared with placebo/no support [3]. Therefore, if we assume that 65% of would‐be varenicline users switch to NRT/bupropion and 35% do not use another medication or an e‐cigarette, this means that the disruption in supply of varenicline has resulted in between ~3900 [(39 600 × 0.65 × 0.069) + (39 600 × 0.35 × 0.15)] and ~8400 [(85 800 × 0.65 × 0.069) + (85 800 × 0.35 × 0.15)] fewer people stopping smoking for at least 6 months each year.
10. Of people who successfully quit smoking for 6 months, approximately 50% will relapse in the long‐term (i.e. after 6 months) [20]. This means that the disruption in supply of varenicline has resulted in between ~1950 (3900 × 0.5) and ~4200 (8400 × 0.5) fewer long‐term (> 6 months) ex‐smokers each year.
11. For the median age of treated people who smoke (aged approximately 40–45 years) [21], there is a 90% risk reduction in 50% excess mortality for long‐term cessation [22]. This means that the disruption in supply of varenicline has resulted in between ~880 (1950 × 0.9 × 0.5) and ~1890 (4200 × 0.9 × 0.5) avoidable deaths each year.
12. Looking beyond 2022, if we assume that smoking prevalence and the rate of quit attempts do not change substantially, varenicline does not return to the market and is used by 0% of people who smoke in a quit attempt, and 35% of those who would have used varenicline if it were available do not use another medication or an e‐cigarette, we therefore estimate that the absence of varenicline could result in up to ~8400 fewer people stopping smoking for at least 6 months, ~4200 fewer long‐term ex‐smokers and ~1890 more avoidable deaths each year.

**FIGURE 2:**
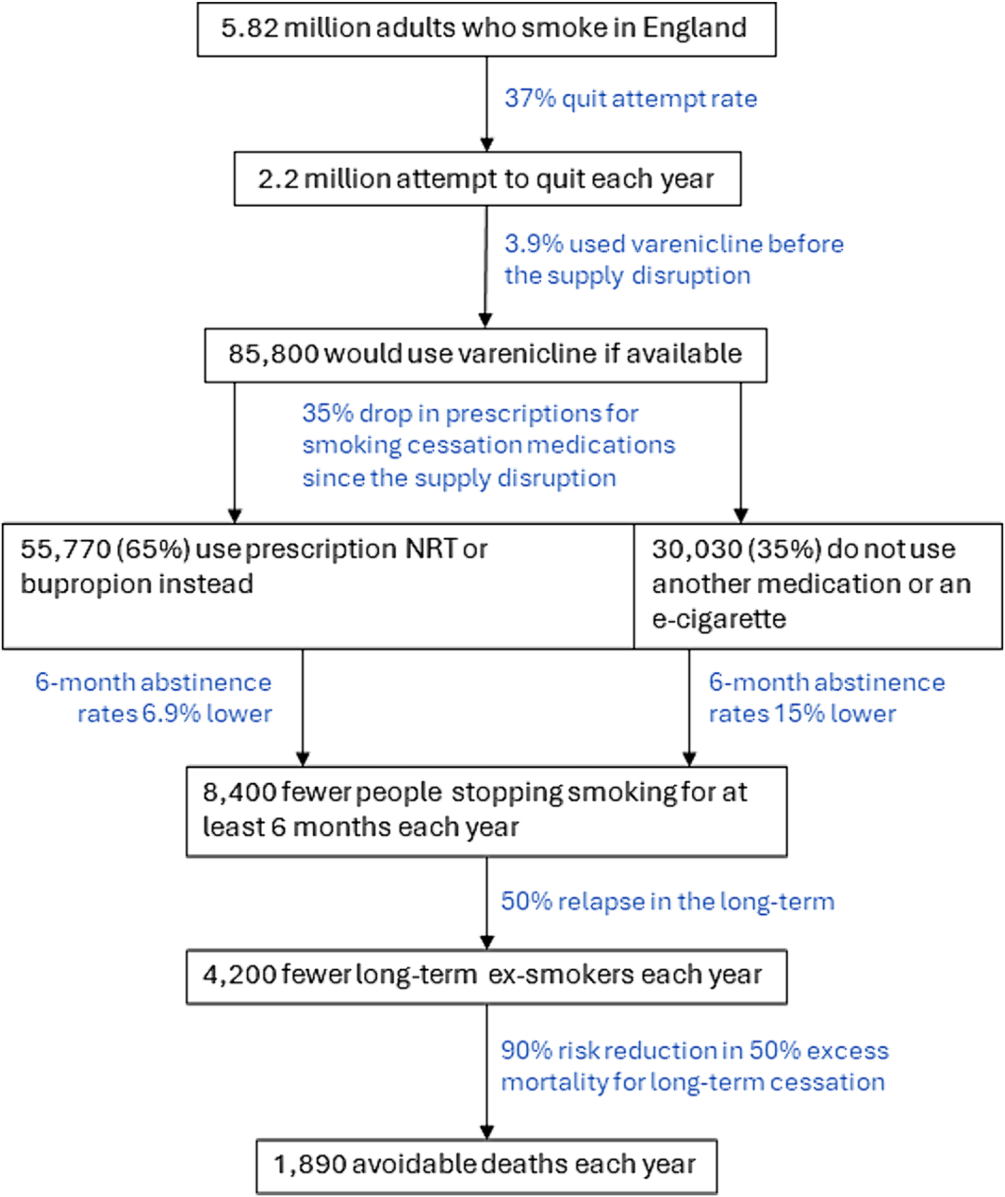
Estimation of the population‐level impact of the varenicline supply disruption on smoking cessation in England. See Results for a more detailed description.

Figure 2 provides a visual summary of our estimation.

## DISCUSSION

Before the varenicline supply disruption in July 2021, the proportion of quit attempts that used varenicline was stable at approximately 3.9%. Since the disruption, unsurprisingly, the trend in varenicline use has changed sharply, with prevalence falling by 69.3% per year since July 2021 to 0.8% of past 6‐month quit attempts in December 2022 (with unmodelled data showing no participants surveyed in the last quarter of 2022 reported using varenicline in their most recent quit attempt). Convergently, just 0.1% of prescriptions for smoking cessation treatments in December 2022 were for varenicline, indicating use by those who tried to quit (> 90% of whom used no prescription medication) had virtually reached 0%. Assuming that varenicline does not return to the market, we estimate this could result in approximately ~8400 fewer people stopping smoking for at least 6 months, ~4200 fewer long‐term ex‐smokers and ~1890 more avoidable deaths each year at current smoking rates.

The supply disruption resulted from laboratory analysis which identified Chantix tablets to contain 150‐470 ng N‐nitroso‐varenicline impurity per 1‐mg tablet [23]. For reference, the acceptable intake limit is 37 ng of N‐nitroso‐varenicline per day and is considered reasonably safe for humans, based upon life‐time exposure [23]. The Food and Drug Administration (FDA) reports ‘a person taking a drug that contains N‐nitroso‐varenicline at‐or‐below this intake every day for 70 years is not expected to have an increased risk of cancer’ [23]. The standard treatment course for Chantix is 12 weeks (two 1‐mg tablets daily). Based upon the upper limit of exposure (470 ng per tablet), a 12‐week course of Chantix would expose the user to 0.079 mg of N‐nitroso‐varenicline. Assuming that N‐nitroso‐varenicline is similarly carcinogenic to tobacco‐specific nitrosamines (TSNAs), this is equivalent to the exposure of smoking 198 cigarettes (at approximately 400 ng of TSNAs per cigarette [24])—or, at a typical consumption level of 11 cigarettes per day [25], 18 days of smoking. Given our estimate of additional avoidable deaths for England alone from the withdrawal of this life‐saving treatment, there appear to have been unfortunate but substantial unintended consequences of attempts to ‘ensure the safety of the US drug supply’ [26].

In the United States, an FDA‐approved generic varenicline became available shortly after Pfizer recalled Chantix, which has helped to fill some of the void left by Chantix (although use of any varenicline product remains at lower levels than before the recall) [27]. In England, where no generic varenicline product is available, the only alternative prescription treatments have been NRT and bupropion. These treatments are less effective than varenicline, but more effective than no treatment [3]. Prescription data show that the use of prescribed treatments has fallen by more than a third since the varenicline supply disruption [15], which does not suggest that people who would have used varenicline chose to use prescription NRT or bupropion instead. Some people may be attracted to varenicline because they want to want to kick their dependence on nicotine entirely or because they have used NRT in a previous failed quit attempt [28], which may make them less inclined to use prescription NRT in the absence of varenicline. Consistent with this, the prescription data show a notable rise in prescriptions of bupropion (a non‐nicotine treatment) following the varenicline supply disruption but little change in NRT prescriptions [15].

It is not yet clear whether varenicline will return to the market in England (either in the form of Pfizer's Champix or a generic version). If it does not, there are alternative medications that could potentially help people to stop smoking with similar effectiveness. Cytisine is less well‐known but has similar properties to varenicline (i.e. structurally similar to nicotine, acts as a partial agonist at nicotinic acetylcholine receptors) and has been shown to be effective for smoking cessation in a number of trials [29, 30, 31]. Cytisine is licensed [32] and has begun to be supplied in England since January 2024. The need to fill the gap left by varenicline is compounded by news of a disruption to the supply of bupropion (Zyban), which was paused by its supplier GSK in December 2022 following similar concerns about the presence of nitrosamine impurities [33].

Strengths of this study include the large, representative sample and repeat cross‐sectional data collection pre‐dating the timing of the supply disruption. A key limitation of the STS analysis was the lag in data collection on quit attempts, which was in the context of the past 6 months. As a result, models probably overestimate the percentage of people who smoke who used varenicline in any given month since the disruption in supply, and underestimate how quickly varenicline use fell after the disruption. While it was possible, in theory, to look at quit attempts over a shorter period (e.g. during the past month), this would have reduced the sample size substantially which, given that varenicline is only used by a small proportion of quitters, would have introduced substantial imprecision. Rather, we triangulated our STS analysis with prescription data from GP practices in England, which provided a more time‐sensitive measure of the decline in use of varenicline. Another limitation is the reliance upon self‐reports of varenicline use in the STS, introducing scope for recall bias. However, unless recall changed during the study period this should not have affected trends. In addition, our data cover a period that included the COVID‐19 pandemic, which was associated with a rise in quitting activity in England [34, 35] that has been sustained over time [36]. While this may have contributed to a decline in the use of varenicline in quit attempts in the short term, if people were less likely to seek out support for stopping smoking, it is unlikely that this drove the large and prolonged decline we observed. Moreover, we modelled changes in varenicline use in relation to the timing of the varenicline supply disruption specifically, which did not directly correspond with any specific COVID‐19 related events.

Our modelling provides simple estimates of the probable population‐level impact upon quitting and smoking‐related mortality based upon the best available data. We made a number of assumptions about future rates of smoking and quit attempts and use of alternative cessation aids, and our estimates will need to be updated if these change substantially. We assumed that the rate of quit attempts did not change, but it is possible that the unavailability of varenicline may have led some people to postpone their quit attempt (or possibly even being advised to) in the absence of an alternative (e.g. if people prefer to use non‐nicotine pharmacotherapy and bupropion having more contraindications). A previous study has shown that the introduction of new medications such as varenicline coincided with an increase in the rate of quit attempts [37]; if the inverse is true, the sudden withdrawal of varenicline would have the opposite effect. More sophisticated analyses should be undertaken when more data are available to obtain a more detailed view of the consequences of the absence of varenicline for people who smoke in England.

In conclusion, the disruption in supply of varenicline since 2021 has coincided with a substantial fall in use of varenicline in attempts to stop smoking in England. Unless people who smoke are provided with alternative, equally effective forms of support, this is likely to hinder progress towards reducing smoking prevalence and smoking‐related harm.

## AUTHOR CONTRIBUTIONS

**Sarah Jackson:** Conceptualization (equal); formal analysis (lead); investigation (equal); methodology (equal); visualization (lead); writing—original draft (lead). **Jamie Brown:** Conceptualization (equal); data curation (lead); funding acquisition (equal); investigation (equal); methodology (equal); supervision (lead); writing—review and editing (equal). **Harry Tattan‐Birch:** Conceptualization (equal); investigation (equal); methodology (equal); writing—review and editing (equal). **Lion Shahab:** Conceptualization (equal); funding acquisition (equal); investigation (equal); methodology (equal); writing—review and editing (equal).

## DECLARATION OF INTERESTS

J.B. has received unrestricted research funding from Pfizer and J&J, who manufacture smoking cessation medications. L.S. has received honoraria for talks, an unrestricted research grant and travel expenses to attend meetings and work‐shops from Pfizer, and has acted as paid reviewer for grant‐awarding bodies and as a paid consultant for health‐care companies. All authors declare no financial links with tobacco companies, e‐cigarette manufacturers or their representatives.

## ETHICS APPROVAL

Ethical approval for the STS was granted originally by the UCL Ethics Committee (ID 0498/001). The data are not collected by UCL and are anonymized when received by UCL.

## Data Availability

Data are available from the corresponding author on reasonable request.
